# Novel Method for Detecting Coughing Pigs with Audio-Visual Multimodality for Smart Agriculture Monitoring

**DOI:** 10.3390/s24227232

**Published:** 2024-11-12

**Authors:** Heechan Chae, Junhee Lee, Jonggwan Kim, Sejun Lee, Jonguk Lee, Yongwha Chung, Daihee Park

**Affiliations:** 1 Info Valley Korea Co., Ltd., Anyang 14067, Republic of Korea; chai@invako.kr (H.C.); lyjourney@invako.kr (J.L.); jking@invako.kr (J.K.); lllss22@invako.kr (S.L.); 2 Department of Computer Convergence Software, Korea University, Sejong 30019, Republic of Korea; ychungy@korea.ac.kr

**Keywords:** agriculture IT, smart agriculture monitoring, audio-visual data, pig cough detection

## Abstract

While the pig industry is crucial in global meat consumption, accounting for 34% of total consumption, respiratory diseases in pigs can cause substantial economic losses to pig farms. To alleviate this issue, we propose an advanced audio-visual monitoring system for the early detection of coughing, a key symptom of respiratory diseases in pigs, that will enhance disease management and animal welfare. The proposed system is structured into three key modules: the cough sound detection (CSD) module, which detects coughing sounds using audio data; the pig object detection (POD) module, which identifies individual pigs in video footage; and the coughing pig detection (CPD) module, which pinpoints which pigs are coughing among the detected pigs. These modules, using a multimodal approach, detect coughs from continuous audio streams amidst background noise and accurately pinpoint specific pens or individual pigs as the source. This method enables continuous 24/7 monitoring, leading to efficient action and reduced human labor stress. It achieved a substantial detection accuracy of 0.95 on practical data, validating its feasibility and applicability. The potential to enhance farm management and animal welfare is shown through proposed early disease detection.

## 1. Introduction

As investment in the pig industry continues to increase, there is a growing need to maximize profitability and enhance productivity in pig farming. Pigs account for approximately 34% of global meat consumption [1]. According to a joint report by the Organization for Economic Cooperation and Development (OECD) and the Food and Agriculture Organization of the United Nations (FAO), global pork consumption is expected to increase by approximately 11% by 2032, compared to 2023 levels [2].

The most fundamental approach to enhancing productivity in pig farming involves carefully monitoring the pigs’ health status and minimizing disease outbreaks [3,4,5]. Through farm monitoring, abnormal pig behavior can be observed, enabling the early detection of diseases. Such early intervention not only helps reduce the spread of disease but also minimizes the stress experienced by the animals [6,7,8]. Timely treatment can prevent more invasive or painful interventions later. Furthermore, the monitoring system provides valuable insights into pigs’ behavioral patterns and stress levels, allowing farm managers to optimize living conditions and improve welfare standards.

However, the continuous monitoring and management of individual pigs’ health is challenging because of limited farm labor [9,10]. Therefore, an efficient system is required to manage pigs in barns with limited personnel [4]. Unlike large-scale farms in advanced livestock countries, South Korean farms are predominantly small- to medium-sized, often raising pigs in confined spaces to maximize production efficiency [11]. This setup increases stress and disease transmission owing to closer contact [12,13]. Airborne respiratory diseases are a leading cause of mortality in such environments, significantly affecting productivity [13,14], with estimated annual losses exceeding KRW 100 billion in South Korea alone [15].

Various studies have been conducted to minimize the impact of pig respiratory diseases through early detection, focusing on Information and Communication Technology (ICT) and computer science approaches that target coughing, a primary symptom [16,17,18,19,20]. For instance, methods have been proposed to classify coughing in diseased pigs to enable early intervention [16,17,18,19], and techniques have been proposed to detect coughing sounds within specific frequency ranges for early disease identification [20]. These approaches allow for a faster response to disease than traditional veterinary diagnostics.

Previous studies have made significant progress in the early detection of respiratory diseases in pigs; however, most of these studies were conducted in simulated environments, and limitations exist when applied directly to practical pig barns. First, to facilitate analysis, research has primarily focused on detecting or identifying diseases from pretrimmed sounds of pigs coughing [16,17,18]. However, practical pig barns contain constant and loud background noise such as pig vocalizations, footsteps, and ventilation sounds. This continuously noisy environment makes it challenging to isolate and analyze coughing sounds effectively in practical pig barns. Second, relying solely on auditory information makes it difficult to pinpoint the exact location of a diseased pig accurately, which hinders early intervention [16,17,18,19]. Without precise location data, extensive measures, such as disinfecting the entire farm or quarantining all pigs, may be required, leading to significant resource and time inefficiency. In extreme cases, the failure to identify the source could result in the culling of the entire pig population in the barn [21].

To address these limitations, this study proposes a system that monitors coughing pigs within a barn using continuous audio and video data. This system detects coughing events across all health stages, as coughing is a common symptom of respiratory diseases, regardless of the disease’s progression. The proposed system comprises three main modules: (1) cough sound detection (CSD), (2) pig object detection (POD), and (3) coughing pig detection (CPD). The CSD module detects coughing intervals using only audio data from a barn, building on research in sound event detection (SED) [22]. Next, the POD module applies object detection techniques to the video data during the detected coughing intervals to identify candidate pigs. Finally, drawing from active speaker detection (ASD) [23,24], the CPD module combines audio and video data to identify a coughing pig.

The proposed system robustly detects pig coughs from continuous audio streams containing various background noises rather than relying on the pretrimmed sound data typically used in laboratory prototypes. Additionally, it identifies the location of the cough, enabling the detection of specific pens or individual pigs as the disease source. This integrated approach, which utilizes audio and video data, offers a more precise and effective solution for the early detection and management of respiratory diseases in pigs. The system allows 24 h monitoring with minimal labor, enabling the early identification of respiratory issues through cough frequency analysis and other advanced techniques [25]. Ultimately, this technological advancement will be crucial in enhancing productivity in the pig industry, minimizing economic losses, and improving animal welfare.

The contributions of this paper are as follows:

1. Advanced Cough Detection System: This paper proposes an advanced system for accurately detecting cough events within pig barns. By employing cutting-edge audio processing techniques, the system ensures the precise identification of cough sounds.
2. Accurate Pig and Pen Identification via Audio-Visual Integration: The proposed system integrates audio and video data to identify coughing pigs and their locations within a barn accurately. This combined approach enables a comprehensive analysis of visual and auditory information to facilitate the effective management of infected pigs.
3. Lightweight and Efficient Monitoring System: By primarily utilizing small-capacity audio data, the system offers a lightweight and efficient monitoring solution, reducing computational load and enhancing processing speed compared to systems that rely solely on video data.

## 2. Related Work

As the significance of respiratory diseases in pigs has been increasingly recognized, researchers have undertaken various engineering studies to detect pig coughing. For instance, researchers have utilized fine-tuned AlexNet to classify pig coughs [16], developed early warning systems using DenseNet-121 with SENets [17], and improved cough classification accuracy by fusing multiple features instead of relying on a single feature [18]. However, these studies were based on trimmed sound data and encountered limitations in processing continuous sound streams in practical farming environments.

Studies that have overcome these limitations have focused on the detection and classification of coughs using continuous sound data. For example, one study [19] employed a pipeline structure that collected sound for anomaly detection. This study utilized an ACAM-based VAD [26] technique to detect the endpoints of coughs and subsequently classified the types of coughs using MnasNet [27]. Although this approach addressed certain existing limitations, it failed to accurately identify the location of the sound-producing object.

Recently, significant progress has been made in sound event detection (SED) research, which focuses on detecting specific sound events from untrimmed audio and pinpointing their locations. For example, several studies have reduced inference time using TCN models, whereas others have developed efficient localization techniques using spatial features [28,29]. However, most studies relied on obtaining stereo sounds, which can be expensive to implement in pig barns. Instead, it is more practical to combine audio data with video information from cameras already installed in barns for efficient management.

However, studies that combine pig coughing sounds with video information are rare. One representative study detected coughs from barn audio, while simultaneously identifying the coughing pigs using video data [20]. This study employed a rule-based algorithm for cough detection and utilized Motion History Images (MHI), a traditional image-processing technique, for pig identification. However, this approach failed to comprehensively record either audio or video semantic information, thereby limiting its ability to capture the diversity of sound and movement patterns. Therefore, a more sophisticated analysis method using deep learning techniques is needed to capture accurate semantic information regarding pig coughs.

Active speaker detection (ASD) is a critical task that utilizes semantic information about movements in videos to locate an object. Research on ASD includes studies on active speaker context (ASC), which focuses on modeling audio-visual information for single speakers [30]; TalkNet, which incorporates audio-visual cross-attention mechanisms and self-attention mechanisms to capture long-term speaking evidence [31]; and frameworks that integrate contextual information among candidates to account for relationships [32]. These studies typically focused only on a few objects (usually three). However, because of the numerous pigs within the barn area, most of these studies are difficult to apply directly to our research.

Consequently, the proposed system detects pig coughing intervals using the SED-CRNN model [33], which specializes in mono-sound detection within the SED research domain. For pig localization, the system incorporates elements of the TalkNet model [31], which is designed to be unaffected by the number of objects. Table 1 summarizes recent engineering studies on pig cough detection compared to the proposed method. Unlike in previous studies, our system detects pig coughs using untrimmed audio data and identifies coughing pigs using video data. Additionally, it identifies coughing pigs within the video’s regions of interest (RoIs). The integration of audio-visual data can improve the accuracy of detecting coughing pigs compared to using only a single type of data by cross-referencing auditory and visual cues. This approach helps reduce the prevalence of false positives, which commonly occur in systems applied to real farm environments where audio-visual noise is abundant. To our knowledge, this is the first study to use deep learning methods to detect and localize coughing pigs using untrimmed audio and video.

## 3. Proposed Method

**Figure 1:**
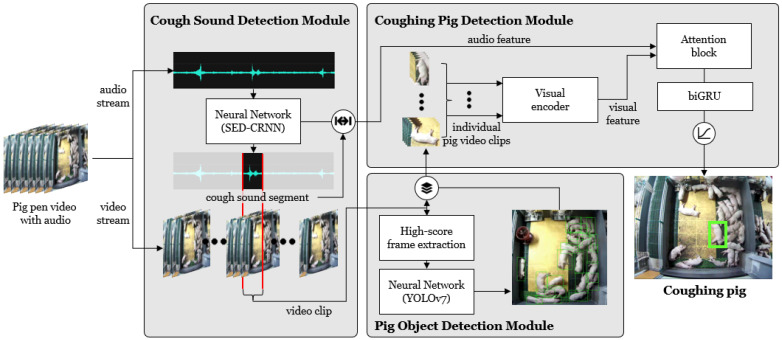
System architecture. The system comprises three modules: a cough sound detection (CSD), a pig object detection (POD), and a coughing pig detection (CPD).

Figure 1 illustrates the structure of the proposed coughing pig detection system. The system comprises three main modules: the CSD module, which detects pig coughs from the sound stream; the POD module, which detects all potential coughing pigs in the video corresponding to the cough intervals; and the CPD module, which integrates both video and audio information to accurately pinpoint a specific pig in the video.

### 3.1. Coughing Sound Detection Module

**Figure 2:**
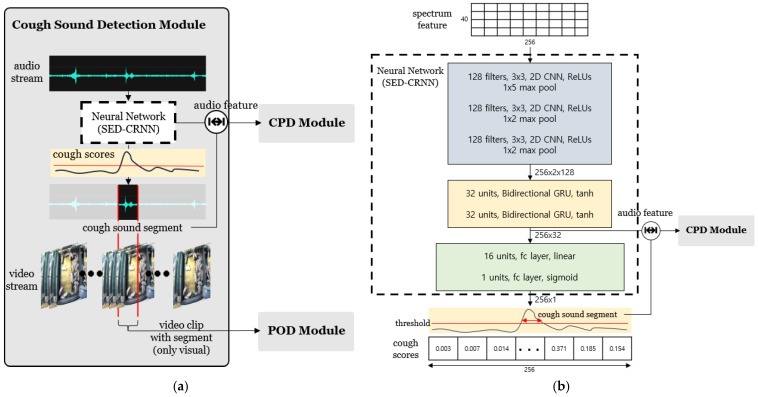
CSD module. (**a**) provides an overview of the overall structure of the CSD module, while (**b**) illustrates the specific architecture of the sound-based cough detection model used within the CSD module.

Figure 2a illustrates the first component, which was designed to detect coughing pigs. It primarily detects pig coughing sounds by using continuous audio data recorded in a pigsty. The neural network model in the CSD module is constructed by modifying the fully connected FC layer of the SED-CRNN model, as shown in Figure 2b. Spectral features are extracted from the audio data and used as inputs for the model. The input data are preprocessed into spectrum data and undergo sequence analysis through a CNN block to extract features from the audio data and pass through the GRU block for temporal data analysis. Finally, two FC layers output probability values (cough scores), indicating whether a cough occurred at each time point. This module identifies coughing events as intervals with scores exceeding a specific threshold. It then matches the corresponding video frames and passes them to the POD module. In addition, the audio features generated during the analysis by the neural network model in the CSD module are passed to the CPD module for audio-visual analysis to identify the pigs that cough. This process uses the vector immediately before the last two FC layers as an audio feature. This approach prevents redundant feature extraction in the CPD module, thereby enhancing the system’s overall efficiency.

### 3.2. Pig Object Detection Module

**Figure 3:**
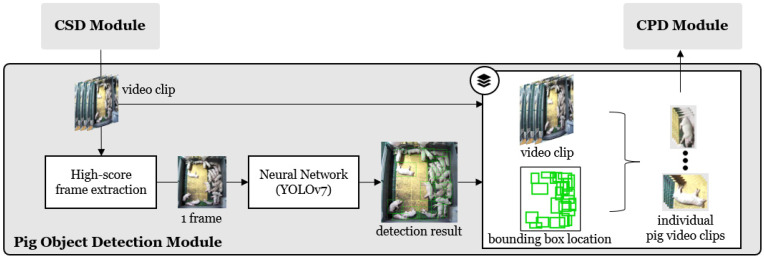
POD module. High-score frames are extracted from the video clip to detect pig objects, and pig-specific crop frames are created based on the detected regions.

**Figure 4:**
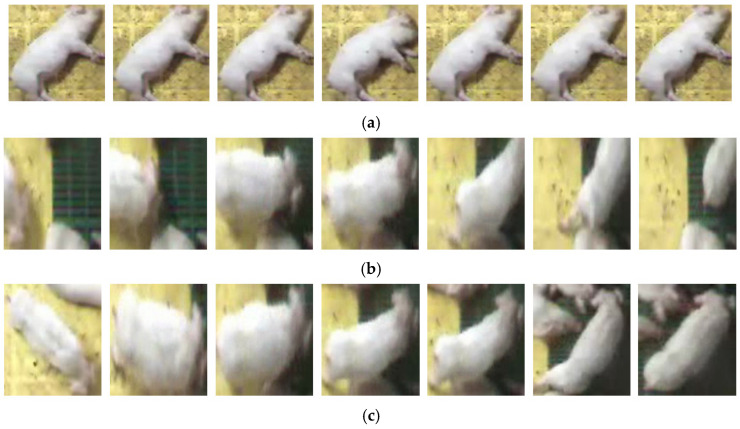
Differences in individual pig videos cropped according to the bounding box. (**a**) A coughing pig using the high-score bounding box (HB), (**b**) a moving pig using the HB, and (**c**) a moving pig without using the HB.

As shown in Figure 3, the POD module separates individual pig objects from the video clips received from the CSD module and passes the corresponding images to the CPD module. Initially, the module extracts a single frame from the video clip and detects the pig object in that frame. The neural network model in the POD module utilizes YOLOv7 [34]. Based on the detected bounding boxes, cropped images of individual pig objects are extracted from the clip (see Figure 4a) and forwarded to the CPD module. The module selects the frame with the highest cough score within the clip from the CSD network’s results. This process ensures that the frame that captures the moment of coughing is selected most accurately. The equation used for selecting a high-scoring frame is as follows.

(1) $$I_{hs}=I_{\arg\max C(t)}$$

The term $I_{hs}$ refers to a high-scoring frame image from a video clip. Here, arg⁡max⁡C(t) indicates the time t at which the cough score C(t) reaches its maximum value. Expressed differently, the process analyzes the cough score C(t) over time to identify the moment with the highest score and extracts frame I corresponding to that exact moment.

The reason why a single bounding box from the high-score frame can be used to crop individual subjects in the clip is that the coughing pigs, which are the focus of this system’s analysis, do not move. A fixed bounding box in one frame is sufficient to capture the visual characteristics of a coughing pig across all frames, as shown in Figure 4a, preserving most of the visual information.

However, for moving subjects, as depicted in Figure 4b, a single bounding box is insufficient for capturing the visual information completely. However, because these moving pigs are not the focus of the analysis, the inability to fully capture their visual information did not affect the research objective. Figure 4c shows the results of tracking the same pig shown in Figure 4b across all frames, capturing the total visual information. However, this method requires detection and tracking for every frame, leading to an increased computational load and slower processing speed. Therefore, targeting only nonmoving pigs and using a high-score frame is more efficient in our system, reducing unnecessary computations and enhancing system performance, as demonstrated in Section 6.2.

### 3.3. Coughing Pig Detection Module

**Figure 5:**
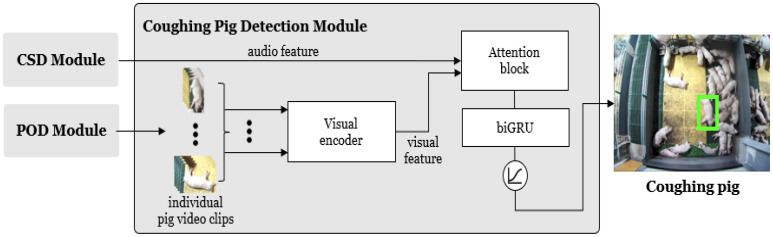
Coughing pig detection module. The CSD module ultimately combines audio information with the visual information from the POD module to detect coughing pigs within the area of interest.

This module receives audio features from the CSD module and cropped images from the POD module and is responsible for identifying the coughing pig among the individual pig objects (Figure 5). Specifically, it comprises a visual encoder to extract the visual characteristics of each object and an attention module combined with a biGRU model to integrate audio-visual features and accurately determine which pig is coughing.

#### 3.3.1. Visual Encoder

**Figure 6:**
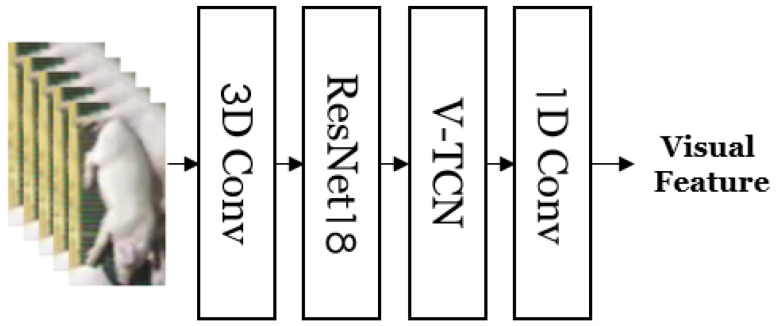
Visual encoder. Visual features are extracted from the cropped pig object videos.

In the visual encoder, the cropped video of the pig object provided by the POD module is used as the input to generate an embedded feature vector containing the movement information of a specific pig. Figure 6 shows the structure of the visual encoder. Initially, the input video undergoes visual feature extraction using 3D convolution layers and ResNet18 [35]. Subsequently, the module extracts the temporal information of the video using a modified version of the Temporal Convolutional Network (TCN) model [36], known as Video-TCN (V-TCN) [31], which has demonstrated strong performance in handling sequential data. Finally, the feature size is adjusted through a 1D convolution layer to align it with the size of the audio features.

#### 3.3.2. Attention Block

**Figure 7:**
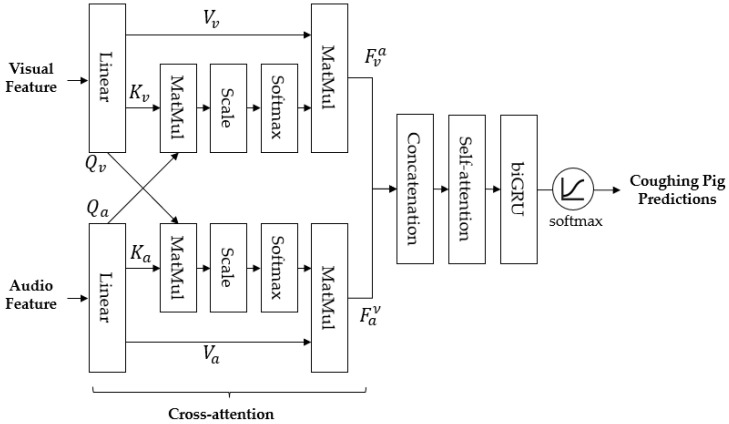
Attention block. It determines the activity status of pigs using attention and the GRU structure with their audio and visual features.

Figure 7 shows a simplified overview of the attention block used in our system. This block combines visual features (from the visual encoder) and auditory features (from the CSD module) to detect coughing pigs. The cross-attention mechanism helps align the audio and visual data by exchanging key information between the two, which ensures both types of features are analyzed together. The combined features are then passed through a self-attention network, which helps the model focus on important patterns that are indicative of coughing behavior. Afterward, these features are processed using a biGRU model [37], which is designed to analyze sequences of data. This allows the system to capture the temporal nature of coughing sounds and movements, improving the accuracy of pig detection. Finally, the model calculates the likelihood that a pig is coughing, and this probability is compared to a threshold to make the final decision. This module mathematically represents the process as shown in the following equations.

(2) $$Attention(Q,K,Q)=\sigma \left(\frac{Q{\left(K\right)}^{T}}{\sqrt{d}}\right)V$$

(3) $$F_{a}^{\nu }=Attention(Q_{v},K_{a},Q_{a})$$

(4) $$F_{v}^{a}=Attention(Q_{a},K_{v},Q_{v})$$

(5) $$F_{av}=F_{a}^{v}⊕F_{v}^{a}$$

(6) $$P=\sigma \left(biGRU\left(Attention(Q_{av},K_{av},Q_{av})\right)\right)$$

Equation (2) represents the basic self-attention mechanism [28], while Equations (3) and (4) illustrate the cross-attention mechanism, where the query ($Q_{a}$, $Q_{v}$) is exchanged to calculate attention for the purpose of sharing audio-visual features. The key ($K_{a}$, $K_{v}$) and value ($V_{a}$, $V_{v}$) are used in the same manner as in basic self-attention. The generated features ($F_{a}^{\nu }$, $F_{v}^{a}$) are then concatenated as shown in Equation (5), and subsequently processed through self-attention, biGRU, and softmax (σ) in sequence, as described in Equation (6), to produce the probability value P.

## 4. Data

This section provides a description of the datasets and preprocessing strategies used in our experiments to validate the proposed system’s CSD, POD, and CPD modules. Each module utilizes datasets tailored to specific objectives, collected from actual pig farm environments. These datasets reflect the real-world challenges encountered in pig barns, including environmental variability such as changes in lighting, pig activity levels, and the presence of farm workers, as well as background noise from both pigs and farm machinery. These variations ensure that the system’s robustness is tested under diverse and realistic conditions. The following sections describe the datasets used for each of the three modules in more detail.

### 4.1. Pig Coughing Sound Dataset

First, mono-sound audio data collected from an actual pig farm at a sampling rate of 16,000 Hz were used to evaluate the CSD module. The audio data, averaging approximately 20 s long and containing at least one instance of a pig coughing, comprised 546 trimmed audio samples. Each audio sample was annotated with the start and end times of the pig’s cough and recorded to millisecond precision (three decimal places). The annotation format followed the strong-label dataset structure provided by DCASE Task4 [22]. In our experiments, we used 420 samples for training and 126 for testing. Each audio sample was converted into a spectrum and preprocessed by dividing it into segments of 256 units with 40 mel-scale dimensions.

### 4.2. Video Footage for Pig Detection

Video footage was used to evaluate the pig detection performance of the POD and CPD modules. To collect this dataset, a camera was deployed on the ceiling of the pigpen, approximately 3 m above the floor, capturing RGB video at a resolution of 640 × 480 pixels and 10 FPS over three days. The main goal of the CPD module is to detect coughing pigs; therefore, we obtained synchronized audio when collecting pig videos.

Subsequently, images were extracted from the collected video, producing 917 images from different scenes, to assess pig detection performance in the POD module. During preprocessing, we manually labeled the given images with bounding boxes around the pig objects. For training, 800 images were used, and 117 images were reserved for validation and resized to 640 × 640 pixels.

### 4.3. Audio-Visual Dataset for Pig Coughing

The CPD module was designed to detect coughing pigs using audio and video sources. Therefore, we employed the same data used for pig detection in the video footage, but these data include audio for 0.5 s before and after the detected coughing events. Additionally, as shown in Figure 4a,b, the data were used to create an audio-visual dataset by cropping them based on the bounding boxes detected by the POC module in the visual component. Notably, audio could not be separated for each pig; therefore, each pig video clip was shared with the same coughing audio. Since there are a total of 23 pigs appearing in the current video, we obtained 23 individual audio-visual datasets for each of the 193 video clips. Since this process is based on the data used in the previous steps, the video maintains a frame rate of 10 FPS, and the audio is kept at a 16,000 Hz sampling rate.

Owing to the characteristics of the collected data, 22 of the 23 pigs in each clip were non-coughing, whereas only one was a coughing pig. This imbalance could lead to a bias toward non-coughing pigs during training. Therefore, the system selects the coughing pig and the two most active non-coughing pigs, resulting in three cropped video segments per clip. Pig activity was determined using the Structural Similarity Index Measure (SSIM) [38], with the movement (Mov) metric calculated as follows:

(7) $$SSIM\left(x,y\right)=\frac{\left(2\mu_{x}\mu_{y}+c_{1}\right)\left(2\sigma_{xy}+c_{2}\right)}{\left(2\mu_{x}^{2}+\mu_{y}^{2}+c_{1}\right)\left(\sigma_{x}^{2}+\sigma_{y}^{2}+c_{2}\right)}$$

(8) $$Mov=1-min\left(SSIM\left(I_{i},I_{\left\lceil L/2\right\rceil }\right)\right),\;\;1\leq \mathrm{i}<L∕2$$

In Equation (7), x and y represent the two images being compared. The parameters μ and $\sigma^{2}$ refer to the average and the variance, respectively, while $\sigma_{xy}$ is the covariance between x and y. Constants $c_{1}$ and $c_{2}$ are introduced to stabilize the division when the denominator is small. Using these, the system compared the SSIM indices of all the frames in the cropped video, particularly between the ith frame and the L/2th frame, as shown in Equation (8). Because an SSIM index closer to 1 indicates minimal differences (i.e., less movement) between frames, the movement index (Mov) was calculated for convenience by subtracting SSIM from 1. Consequently, a higher Mov value indicates more significant movement in the video.

The Mov value was calculated for all 22 non-coughing pigs, and the two pigs with the highest Mov scores were selected for inclusion in the training along with the coughing pigs. Consequently, 193 video clips were divided into 173 video clips for training and 20 for validation. The actual training involved 519 audio-visual datasets (173 clips × 3 pigs), whereas the validation used 460 datasets (20 clips × 23 pigs). The cropped visual data contained pigs of varying sizes, so each image was of a different size. Therefore, all the images were resized to 112 × 112 grayscale images. For the audio data, we used the features from the CSD module. After undertaking the same preprocessing steps described in Section 4.1, we utilized the 128-dimensional feature vector obtained from the CSD module.

## 5. Results

This section evaluates the proposed method’s performance in detecting coughing pigs using real-world audio and video data collected from pig farms. The deep learning networks used in the CSD, POD, and CPD modules were trained for this purpose. The experiments were conducted using a GeForce RTX 2070 8 G GPU. For data processing in this system, it is recommended to use a server with at least 4 GB of GPU memory. Additionally, PyTorch 1.3.0 was used during the testing phase.

### 5.1. Experimental Design

The proposed system comprises deep learning networks designed to achieve three primary objectives: cough sound detection (CSD module), pig object detection (POD module), and coughing pig detection (CPD module). Table 2 provides a detailed overview of the purpose, evaluation metrics, dataset types, and hyperparameters of each experiment. This systematic experimental design objectively assessed the performance of each module and ensured that the applicability of the system in real farm environments could be thoroughly validated.

### 5.2. Results for CSD Module

The performance of the neural network model in the CSD module was evaluated using an Event-based F-score [39]. The experimental results demonstrated a cough detection F-score of 0.8387. The results indicated a high level of detection accuracy, even when compared with existing results in the field of sound event detection [22], suggesting the effectiveness of the model in real-world farm environments with complex sound events.

**Figure 8:**
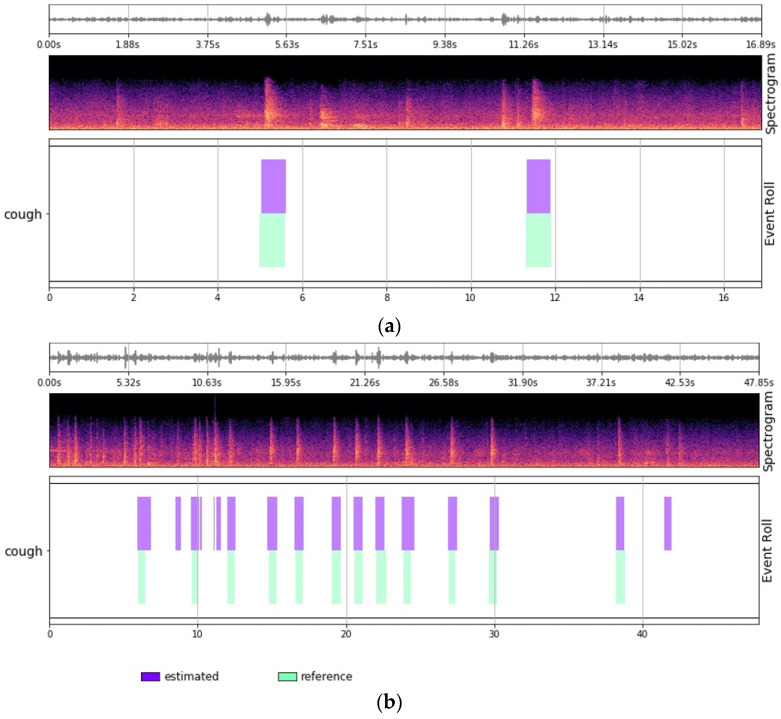
Panels (**a**,**b**) qualitatively show the sound detection results in different environments. The label ‘estimated’ is the cough area predicted by the model’s CSD module, and ‘reference’ corresponds to the ground truth.

Figure 8 provides the qualitative results, showing the accurate detection of cough intervals from real farm sound data (Figure 8a) and the model’s ability to detect cough sounds in a noisy environment (Figure 8b). These findings confirm the capability of the model to detect and analyze cough sounds efficiently in actual farm settings.

### 5.3. Results for POD Module

**Figure 9:**
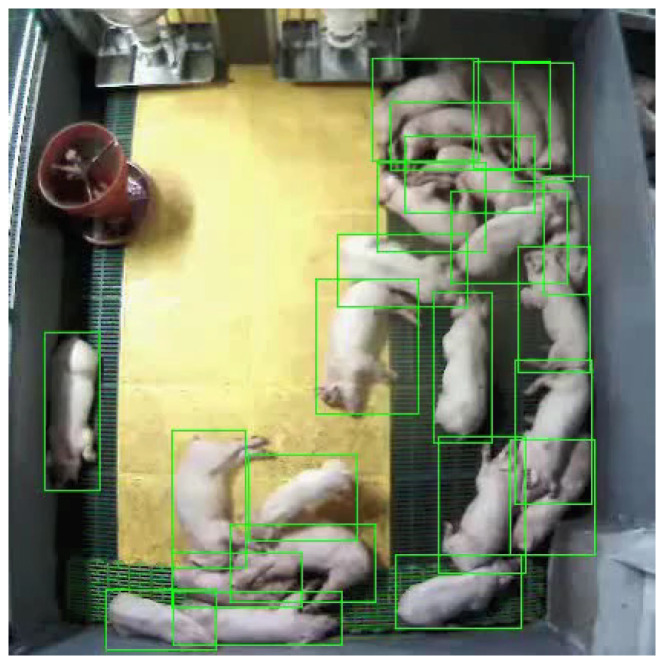
Qualitative results of neural network model in POD module (YOLOv7).

The model was evaluated with the mAP metric [41], which is widely used in object detection tasks. The experimental results yielded an impressive mAP of 0.986, indicating that the model correctly detected almost all the coughing pigs. Figure 9 shows the qualitative results of the neural network model in the POD module.

**Figure 10:**
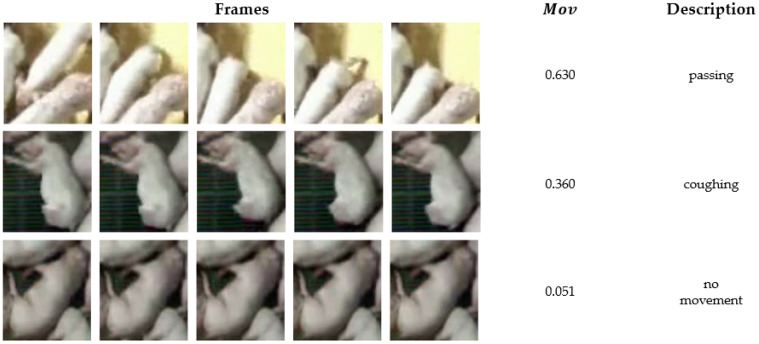
Mov results of pig object cropped videos according to the situation.

Figure 10 presents a sample of the Mov measurement results calculated to select the objects to be used in training. The “Frames” in the Figure display the changes in the cropped video over time, with the lower rows indicating videos with less movement. The first video shows a pig passing through the screen; the second shows a struggling pig; the third depicts a coughing pig; the fourth shows minor movements in the background; and the final video shows a pig with no movement. The Mov values decreased proportionally with the magnitude of movement observed in each video. The results confirm that the Mov measurement based on SSIM accurately reflects the actual movement of the pig objects.

### 5.4. Results for CPD Module

In this section, we integrate the results from the CSD and POD modules to analyze the performance of the CPD module quantitatively and qualitatively. For the quantitative analysis, the metrics included precision, recall, F1-score, clip accuracy, and top-1 clip accuracy. Precision, recall, and F1-score were used to evaluate individual objects and determine the correctness of predictions for each of the 460 objects across 20 validation videos. In contrast, clip accuracy assesses the accuracy at the level of all clips. Because each clip contained a single pen, predictions were considered correct only when the model accurately predicted all objects within the pen. For instance, if Pig 3 is the actual coughing pig but the model predicts Pigs 3 and 9 as coughing, the result is deemed incorrect (as Pig 9 was incorrectly predicted). This concept can be mathematically expressed as follows:

(9) $$S=\left\lfloor \frac{1}{N}\sum_{\dot{i}=1}^{N}Y_{i}\right\rfloor$$

(10) $$Clip\;accuracy=\frac{1}{M}\sum_{i=1}^{M}S_{i}$$

In this context, N represents the number of pigs within the pen (23 in this experiment); i.e., N = 23. $Y_{i}$ indicates whether the ith pig is correctly predicted, assigning a value of 1 for a correct prediction and 0 otherwise. Consequently, the score S in Equation (9) is 1 only if all pigs in the clip are correctly predicted; otherwise, it is 0. Equation (10) calculates the overall clip accuracy, where M represents the number of clips used for the accuracy measurement. This evaluation metric is stricter than the individual object metrics and provides a more rigorous assessment of the model’s performance. Next, top-1 clip accuracy adjusts the clip accuracy by selecting only the pig with the highest confidence score as the coughing pig when multiple pigs are predicted, reflecting the system’s characteristic that there can only be one actual coughing pig per detection event in the CSD module. This clip-level performance metric allowed for a more stable performance evaluation in a pen environment with an imbalanced 22:1 class ratio by selecting the most likely coughing pigs.

The proposed model’s performance is presented in Table 3, where it demonstrates satisfactory results with an F1-score of 0.77 and a clip accuracy of 0.6. Notably, the model achieved a high top-1 clip accuracy of 0.95, effectively identifying coughing pigs within the clip. To ensure the robustness of the results, the performance metrics were averaged over five independent experimental runs, and the standard deviation (±) is provided in the table to demonstrate that the model’s performance remains stable under various conditions. Various comparative experiments were conducted to validate the performance of the model further, and the detailed results of these experiments are discussed in subsequent sections.

## 6. Ablation Study

### 6.1. CSD Audio Feature

**Figure 11:**
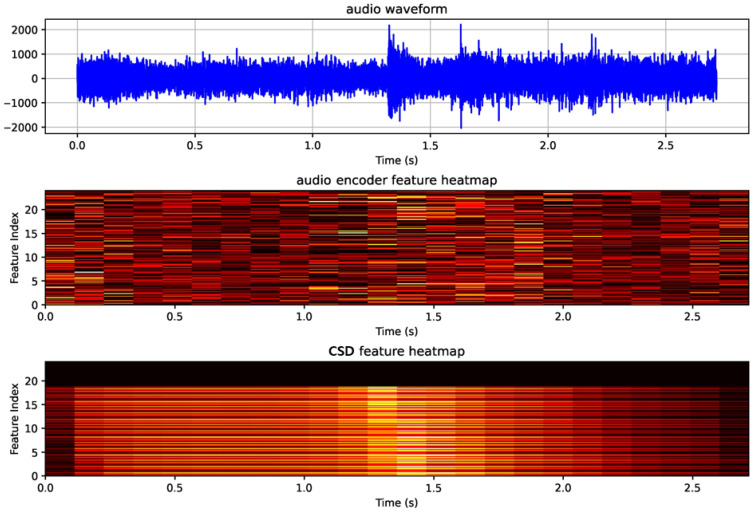
Qualitative comparison of the feature representation results of the CSD module and the audio encoder’s feature representation results.

The CPD module utilizes the audio features extracted by the CSD module, by employing the CA method. For comparative evaluation, another model without CA used TalkNet’s audio encoder to generate audio features. As shown in Table 3, the proposed model with CA produced an F1-score improvement of 0.07, indicating that the audio features of the CSD module accurately captured the input audio characteristics. Figure 11 shows that the CSD module captured the coughing event (approximately 1.3 s) better than the audio encoder. Employing the CA method also improved processing speed and memory efficiency by eliminating the audio encoder process. Table 4 highlights the significant improvements in processing speed and parameter count when applying the CA method, thus confirming its advantages regarding both detection performance and computational efficiency.

### 6.2. High-Score Box

In Table 3, “HB” denotes use of the high-score frame box, as illustrated in Figure 4. When HB is not applied, the object detection and tracking tasks must be performed on every clip frame. For comparison, DeepSORT [47] was used as the tracking method when HB was not used. The results in Table 3 show significant performance improvements when using HB. Specifically, Models 3, 4, and 7 and the proposed model achieved a recall of 0.95, nearly perfectly detecting coughing pigs. Without HB, object detection is performed in every frame. This leads to inconsistencies in the bounding box size, which confuse the interpretation of the objects’ movement information and negatively affects learning. Table 5 compares the movements of the stationary and coughing pigs (Mov). When HB was applied, the movement difference was quantified, whereas, in the absence of HB, the difference was less pronounced. In summary, the HB method stabilizes the box location and effectively captures and interprets the movement information of pigs, leading to higher accuracy.

**Figure 12:**
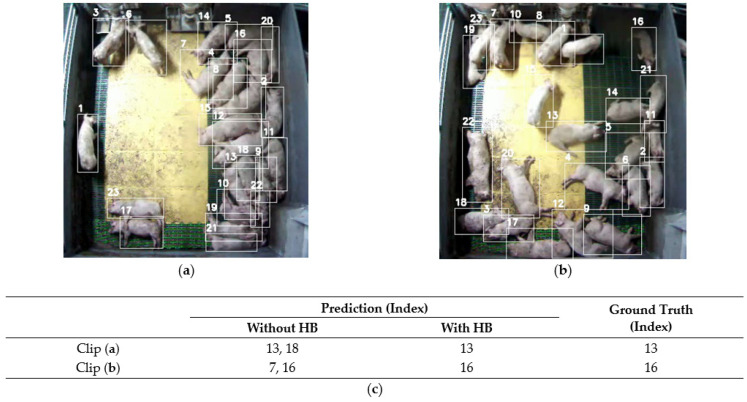
Qualitative results of applying the HB method. (**a**,**b**) are still shots of video clips in different situations, where the numbers indicate the index of the predicted pig. (**c**) shows the coughing pig prediction results by applying the HB method.

Figure 12 illustrates the qualitative results of the CPD module with and without HB application. Figure 12a,b show still shots from clips representing different pen scenarios. Figure 12c shows the ground truth and predicted outcomes for the coughing pigs identified using the proposed system. As shown in Figure 12a, when HB is applied, the system correctly identifies Pig 13 as coughing. Without HB, however, it incorrectly identifies Pig 18, which is adjacent to Pig 13, likely because Pig 13’s movement impacts Pig 18’s bounding box. Similarly, in Figure 12b, Pig 7 is mistakenly identified as a coughing pig without HB, owing to the movement caused while feeding. These results demonstrate that applying the HB method enhances the robustness of the system in detecting coughing pigs, even with movement changes.

In addition, Table 5 illustrates the processing time differences in the POD module depending on whether the HB method is applied. With HB, object detection occurred only once. By contrast, without HB, every clip frame underwent detection, and processing took approximately 20 times longer. Consequently, as did the CA method, the HB method significantly enhanced both speed and accuracy, demonstrating its positive impact across all aspects of the system.

### 6.3. biGRU

In the CPD module, biGRU was used to determine whether a pig was coughing (GRU method in Table 3). For comparison, an FC layer was used when biGRU was not applied. The experimental results showed improved performance in models in which stable training was achieved, excluding Models 1 and 2. Because biGRU is an RNN-based model specialized in sequential data analysis, it was well suited for this study. Although the use of biGRU increases processing time and the number of parameters compared to the FC layer, the performance gains justify its use.

## 7. Discussion

### 7.1. Visual Only vs. Audio-Visual Feature

This experiment aimed to validate the effectiveness of combining audio-visual features to detect coughing pigs. The results in Table 6 demonstrate that even using visual features alone achieved some accuracy, indicating that visual features contain helpful information for detecting coughing pigs. However, the proposed model, which utilizes both audio and visual features, showed evidence of higher accuracy across all metrics than only using visual features. The improvement in performance is particularly noticeable in terms of precision. This can be interpreted as a significant reduction in the false positive issue, which commonly occurs in real pig farm environments, due to the ability to cross-reference auditory and visual cues when both types of information are utilized, as opposed to relying on a single modality. These findings suggest that integrating audio and visual information models the barn environment and pig behavior more effectively, leading to greater detection accuracy.

### 7.2. Outside the RoI

The previous barn experiments were conducted under the condition that coughing occurred within the RoI. Barn-level management allows pigs requiring different treatments in each barn to receive more efficient care. However, even if the camera captures only the RoI, audio data can still capture sounds outside the RoI. Therefore, this experiment aimed to evaluate the ability of the system to identify and filter coughing sounds outside the RoI in a practical barn environment. For this purpose, 200 additional clip datasets were collected in which the coughing occurred outside the RoI. Because there was no ground truth for individual pigs in this scenario, clip accuracy was the only evaluation metric used. The system was considered correct only if all the pigs were identified as non-coughing. Table 7 shows the experimental results, in which the system achieved high accuracy (0.81) in detecting coughing outside the RoI. The results indicate that the system can effectively filter out internal and external coughs using audio-visual data.

### 7.3. Application Results

**Figure 13:**
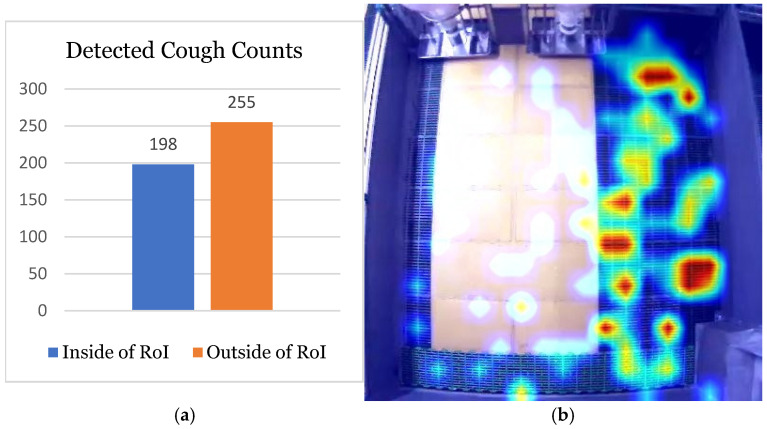
Results obtained by applying the proposed system to actual pig barn data are shown. (**a**) shows the number of cough detections inside and outside the RoI. (**b**) is a heatmap of coughing that occurred inside the RoI, where red indicates areas with higher cough frequency. From this, users can identify areas where coughing frequently occurs.

This section presents the results of applying the proposed system to practical barn data that were not used for training or validation, as shown in Figure 13. The dataset comprised approximately four days of data from a barn housing 23 pigs. Figure 13a shows the number of coughs detected inside and outside the region of interest (RoI). Despite microphone coverage limitations, more coughs were detected outside the RoI, owing to the larger area outside the barn. Distinguishing and recording the number of coughs on a barn-by-barn basis can significantly improve the monitoring of abnormal conditions in specific barns.

In addition, Figure 13b provides a heatmap of cough occurrence within the RoI, with redder areas indicating higher frequencies of coughing. The heatmap revealed that significantly more coughs occurred on the right-hand side of the barn than on the left-hand side. This difference was likely due to more pigs resting on the right-hand side, which was farther from the bright corridor frequently used by farm personnel on the left-hand side. Identifying areas of frequent coughing can be instrumental in analyzing various conditions. This could facilitate targeted responses to coughing events based on specific areas and help identify the causes of coughing by region.

In summary, pig farm owners and managers can make data-driven decisions, respond to health issues early, optimize resource allocation, and enhance farm management by understanding the frequency and distribution of coughing events inside and outside the RoI. Furthermore, the system could be enhanced by incorporating automatic health management actions based on detected coughing events. For example, once the system identifies abnormal coughing patterns, it could trigger actions such as adjusting ventilation, notifying farm personnel, or isolating potentially sick pigs to prevent the spread of disease. These automated responses would reduce the reliance on manual interventions and allow for more efficient and timely management of pig health, improving overall farm productivity and animal welfare. Future research could explore the integration of such automated management actions, further enhancing the system’s role in comprehensive health monitoring.

## 8. Conclusions

In this study, we proposed a novel audio-visual multimodal system for detecting and monitoring coughing pigs in a barn environment. Inspired by SED and ASD tasks, this system was adapted for cough detection in pigs. The system comprises three main modules: the cough sound detection (CSD) module, which detects coughs from audio streams; the pig object detection (POD) module, which identifies individual pigs in the corresponding video segments; and the coughing pig detection (CPD) module, which uses the visual information from the CSD and POD modules to identify a coughing pig. To validate the system, we deployed microphones and cameras in a pig barn to collect audio and video data simultaneously. The experimental results demonstrate that the system efficiently detects coughing pigs with 95% accuracy, confirming its potential for practical application in pig farms.

While the system shows strong performance, future research will focus on three key areas to further enhance its practicality and scalability. First, optimizing the system for real-time data processing is crucial to ensure that the model can operate in real-world farming environments where rapid detection and intervention are essential. Second, the system will be tested under more challenging noise conditions, such as extreme noise overlaps from machinery or simultaneous coughing from multiple pigs in larger-scale farms with hundreds of pigs and multiple barns. This will validate the system’s robustness and applicability in more complex and noisy farm environments. Finally, the integration of an efficient pig-tracking algorithm will allow for individual pig management, improving the system’s capability to monitor and manage each pig’s health more effectively beyond the barn level.

These findings highlight the potential of the proposed system as a vital tool for monitoring pig health and enhancing productivity in pig barns. By effectively monitoring cough occurrence inside and outside the barn, the system enables the barn environment to be comprehensively managed. Given the scarcity of research that integrates audio and visual information for pig farm management, this study makes a significant contribution to the field. This is expected to advance pig farm management practices and improve animal welfare.

## Figures and Tables

**Table 1:** Some recent studies related to pig cough.

Ref.	Data Type	Task	Method	Semantic Information	Recognize Individual Objects	Configure RoI
Yin, et al. 2021 [17]	Trimmed Audio	Classification	Fine-Tuned AlexNet	✔	✘	✘
Song, et al. 2022 [18]	Trimmed Audio	Classification	SE-DenseNet	✔	✘	✘
Yin, et al. 2023 [19]	Trimmed Audio	Classification	Fusion SVM	✔	✘	✘
Hong, et al. 2020 [20]	Untrimmed Audio	Sound Detection and Classification	ACAM-Based VAD and MnasNet	✔	✘	✘
Kim, et al. 2015 [21]	Untrimmed Audio and Video	Sound Detection and Localization	Rule-Based and Image Processing	✘	•	•
Proposed	Untrimmed Audio and Video	Sound Detection and Localization	MultimodalCNN and GRU	✔	✔	✔

**Note**: ✔: Yes, ✘: No, •: Not Specific.

**Table 2:** Experimental design overview for deep learning networks.

Module	Objective	Evaluation Metrics	Dataset Type	Hyperparameters
CSD	To detect cough sound events accurately.	Event-based F-score [39]	Pig cough audio data	300 epochs, batch size: 64, learning rate: 0.001, Adam optimizer [40], binary cross-entropy loss, batch normalization [41] on all CNN layers, 0.5 dropout [42] on all CNN and GRU layers, sigmoid output threshold: 0.7
POD	To detect individual pig objects in images.	mAP [43]	Pigpen image data	200 epochs, batch size: 16, learning rate: 0.01, SGD momentum optimizer [44] (momentum: 0.937), data augmentation: HSV-Hue, HSV-Saturation, HSV-Value, translate, scale, flip, mosaic, mixup [45,46]
CPD	To identify specific coughing pigs accurately.	Precision, recall, F1, clip accuracy, Top-1 clip accuracy	Pigpen video data with both audio and visual components	300 epochs, batch size: 4, learning rate: 0.0001 (decreased by 5% per epoch), Adam optimizer, binary cross-entropy loss, visual data augmentation: random flip, rotate, crop

**Table 3:** CPD module’s accuracy compared against that of different methods.

Index	Method			Precision	Recall	F1	Clip Accuracy	Top-1 Clip Accuracy
	CA	HB	GRU					
1				0.16 ± 0.05	0.55 ± 0.07	0.25 ± 0.07	0.00 ± 0.00	0.15 ± 0.05
2			✔	0.12 ± 0.04	0.45 ± 0.01	0.19 ± 0.06	0.00 ± 0.00	0.05 ± 0.04
3		✔		0.34 ± 0.02	0.95 ± 0.02	0.50 ± 0.02	0.35 ± 0.02	0.80 ± 0.03
4		✔	✔	0.56 ± 0.01	0.95 ± 0.01	0.70 ± 0.06	0.45 ± 0.05	0.90 ± 0.01
5	✔			0.19 ± 0.05	0.90 ± 0.07	0.31 ± 0.04	0.00 ± 0.00	0.50 ± 0.01
6	✔		✔	0.31 ± 0.03	0.75 ± 0.03	0.44 ± 0.04	0.20 ± 0.04	0.45 ± 0.02
7	✔	✔		0.37 ± 0.04	0.95 ± 0.02	0.53 ± 0.04	0.25 ± 0.03	0.75 ± 0.03
Proposed	✔	✔	✔	0.65 ± 0.04	0.95 ± 0.01	0.77 ± 0.03	0.60 ± 0.03	0.95 ± 0.01

**Table 4:** CPD module: processing time and parameter measurement against different methods.

Index	Method		Average Processing Time per Object (s)	#Params.
	CA	GRU		
1			0.027	15.7 M
2	✔		0.013	14.4 M
3		✔	0.031	16.0 M
Proposed	✔	✔	0.018	14.7 M

**Table 5:** Comparative results based on applying HB. ‘Average processing time per clip’ indicates the average time for processing the POD module’s clip image. ‘Mov’ quantifies the average movement for each class.

Method	Average Processing Time per Clip(s)	Mov	
		Coughing Pig	Pig Without Movement
without HB	0.276	0.513	0.349
with HB	0.014	0.388	0.050

**Table 6:** Performance comparison between using only visual features and using a combination of audio-visual features.

Method	Precision	Recall	F1	Clip Accuracy	Top-1 Clip Accuracy
Visual only	0.43 ± 0.06	0.90 ± 0.01	0.58 ± 0.05	0.45 ± 0.04	0.75 ± 0.04
Proposed (Audio-visual)	0.65 ± 0.04	0.95 ± 0.01	0.77 ± 0.03	0.60 ± 0.03	0.95 ± 0.01

**Table 7:** Accuracy in detecting coughs occurring outside of RoI.

	Outside of RoI Clip Accuracy
Proposed	0.81 ± 0.05

## Data Availability

No new data were created or analyzed in this study. Data sharing is not applicable to this article.
